# Supplementary material for: ProteinShader: illustrative rendering of macromolecules
Source: BMC Struct Biol. 2009 Mar 30;9:19. doi: 10.1186/1472-6807-9-19 (PMC2672931; doi:10.1186/1472-6807-9-19)
Supplement: Additional file 1 — ProteinShader program without source code. This compressed file contains the complete ProteinShader program including associated libraries, but no source code. A README.txt file gives an overview of the ProteinShader distribution, and the index.html file in the help subdirectory has directions on getting started with the program as well as a set of tutorials. [file 1472-6807-9-19-S1.zip › ProteinShader-beta-0_9_4-binary/help/api/org/proteinshader/structure/class-use/Bond.html]

Uses of Class org.proteinshader.structure.Bond (ProteinShader API)


|  |  |  |
| --- | --- | --- |
| **Overview** \| **Package** \| **Class** \| **Use** \| **Tree** \| **Deprecated** \| **Index** \| **Help** | |  |
| PREV   NEXT | **FRAMES**    **NO FRAMES**     **All Classes** |  |


---


## **Uses of Class org.proteinshader.structure.Bond**

| Packages that use Bond | |
| --- | --- |
| **org.proteinshader.graphics.adapter** | Holds the StructureToGraphics class, which is used to manage the use of the drawing classes and OpenGL display lists. |
| **org.proteinshader.structure** | Holds the classes that store information from a Protein Data Bank file: Structure, Model, Chain, AminoAcid, Heterogen, Water, Atom, Bond, Helix, BetaStrand, Loop, *etc*. |
| **org.proteinshader.structure.visitor** | Holds Visitor classes that know how to traverse the hierarchy of objects held by class Structure. |

Uses of Bond in org.proteinshader.graphics.adapter

| Methods in org.proteinshader.graphics.adapter with parameters of type Bond | |
| --- | --- |
| `void` | `StructureToGraphics.draw(GL gl, Bond bond)`             Draws the Bond as a single cylinder for a Balls-and-Sticks style display, but two cylinders for a Sticks style display. |

Uses of Bond in org.proteinshader.structure

| Methods in org.proteinshader.structure that return Bond | |
| --- | --- |
| `Bond` | `Atom.getBond(Atom dstAtom)`             Returns the Bond from this Atom (the source) to the destination Atom. |

| Methods in org.proteinshader.structure that return types with arguments of type Bond | |
| --- | --- |
| `Iterator<Bond>` | `Atom.iteratorBonds()`             Returns an Iterator for the Bonds held by this Atom. |

| Methods in org.proteinshader.structure with parameters of type Bond | |
| --- | --- |
| `void` | `Atom.removeBond(Bond bond)`             Removes the Bond if it exists. |

Uses of Bond in org.proteinshader.structure.visitor

| Methods in org.proteinshader.structure.visitor with parameters of type Bond | |
| --- | --- |
| `void` | `ModifierVisitor.visit(Bond bond)`             If a BondModifier has been added, it will be used to modify the Bond. |
| `void` | `Visitor.visit(Bond bond)`             If debug is true, the atomID of both the source Atom and the destination Atom will be printed to standard out. |
| `void` | `SFWriterVisitor.visit(Bond bond)`             Writes all information held by a Bond. |
| `void` | `VisibilityVisitor.visit(Bond bond)`             Tests the visiblity of the Bond in order to determine if it should be transferred to the opaque Atoms and Bonds or translucent Drawables list. |
| `void` | `BondGeneratorVisitor.visit(Bond bond)`             NO OPERATION: This Visitor method is not needed by the bond generator. |

---


|  |  |  |
| --- | --- | --- |
| **Overview** \| **Package** \| **Class** \| **Use** \| **Tree** \| **Deprecated** \| **Index** \| **Help** | |  |
| PREV   NEXT | **FRAMES**    **NO FRAMES**     **All Classes** |  |


---

# *Copyright © 2007-2008*
